# Risk factors for underlying comorbidities and complications in patients with hepatitis B virus-related acute-on-chronic liver failure

**DOI:** 10.1017/S0950268822001169

**Published:** 2022-07-05

**Authors:** Wei-zhen Weng, Jun-feng Chen, Xiao-hua Peng, Miao Huang, Jing Zhang, Jing Xiong, Hui-juan Cao, Bing-liang Lin

**Affiliations:** 1 Department of Infectious Diseases, The Third Affiliated Hospital of Sun Yat-Sen University, Guangzhou, China; 2 Department of Gastroenterology, The Seventh Affiliated Hospital of Sun Yat-Sen University, Shenzhen, China; 3 Department of Nursing, Guangzhou Red Cross Hospital, Fourth Affiliated Hospital of Jinan University, Guangzhou, China; 4 GuangDong Provincial Key Laboratory of Liver Diseases, The Third Affiliated Hospital of Sun Yat-Sen University, Guangzhou 510630, China; 5 Key Laboratory of Tropical Disease Control, Ministry of Education, Sun Yat-Sen University, Guangzhou 510030, China

**Keywords:** ACLF, comorbidity, complication, HBV, mortality

## Abstract

Hepatitis B virus-related acute-on-chronic liver failure (HBV-ACLF) is a severe and life-threatening complication, characterised by multi-organ failure and high short-term mortality. However, there is limited information on the impact of various comorbidities on HBV-ACLF in a large population. This study aimed to investigate the relationship between comorbidities, complications and mortality. In this retrospective observational study, we identified 2166 cases of HBV-ACLF hospitalised from January 2010 to March 2018. Demographic data from the patients, medical history, treatment, laboratory indices, comorbidities and complications were collected. The mortality rate in our study group was 47.37%. Type 2 diabetes mellitus was the most common comorbidity, followed by alcoholic liver disease. Spontaneous bacterial peritonitis, pneumonia and hepatic encephalopathy (HE) were common in these patients. Diabetes mellitus and hyperthyroidism are risk factors for death within 90 days, together with gastrointestinal bleeding and HE at admission, HE and hepatorenal syndrome during hospitalisation. Knowledge of risk factors can help identify HBV-ACLF patients with a poor prognosis for HBV-ACLF with comorbidities and complications.

## Introduction

Acute-on-chronic liver failure (ACLF) is a life-threatening disease that leads to multi-organ failure and high short-term mortality, causing a variety of symptoms including jaundice, coagulopathy, ascites, haemorrhage, cholestasis, hepatic encephalopathy (HE) and hepatorenal syndrome (HRS) [1]. The most common cause of liver failure in Asia is chronic hepatitis B virus (HBV) infection, with a mortality rate of 63–72.3% [2, 3].

The prevalence of HBV in China is 5–6%, which means approximately 70 million HBV carriers in China [4]. Many have comorbidities and prior studies have focused on the impact of comorbidities on patients with HBV-related diseases, such as chronic kidney disease (CKD) [5, 6], diabetes [7] and nonalcoholic fatty liver disease (NAFLD) [8, 9]. As for the complications that occur with HBV-ACLF, some studies suggest a relationship between them. Bacterial infections, HE and HRS, are risk factors for mortality in patients with ACLF [10–12].

There is limited information on the impact of various comorbidities on HBV-ACLF in large populations. The relationship between comorbidities, complications and mortality has not been validated in clinical studies with large samples. Comorbidities are not considered as part of the most widely used prognostic tools (the Child-Pugh and model for end-stage liver disease (MELD) scoring systems).

Based on this background, we designed this retrospective study to analyse the clinical characteristics, complications and mortality of 2166 patients with HBV-ACLF in our hospital and to assess the relationship between them.

## Materials and methods

### Patients

In this study, retrospective observational research was used. All patients were hospitalised at the Third Affiliated Hospital of Sun Yat-sen University, a large tertiary general hospital with more than 40 000 patients discharged annually. We extracted the records of all patients diagnosed with ACLF from 1 January 2010 to 1 March 2018, from the hospital's electronic database of medical records.

The diagnosis of HBV-ACLF was made according to the 2018 Chinese guidelines for diagnosing and treating liver failure [13]. ACLF was characterised by liver injury manifested by jaundice (total serum bilirubin ⩾10 mg/dl) and coagulopathy (international normalised ratio ⩾1.5 or prothrombin activity <40%), complicated by ascites and/or encephalopathy within 4 weeks, as determined by physical examination, in previously diagnosed or undiagnosed chronic patients with liver disease. The exclusion criteria were incomplete information, a hospital stay <24 h and uncertain clinical outcome.

Medical record search strategy: records were searched in the hospital medical record information system for the following diagnostic codes and terms: ‘hepatitis B virus (ICD-10 code: B16.905)’, ‘liver failure (ICD-10 code: K72)’ and ‘liver cirrhosis (ICD-10 code: K74.151)’, or other diagnostic terms for various complications commonly seen in patients with ACLF, such as hepatic encephalopathy (ICD-10 code: K72.903) and hepatorenal syndrome (ICD-10 code: K76.702).

**Fig. 1.**
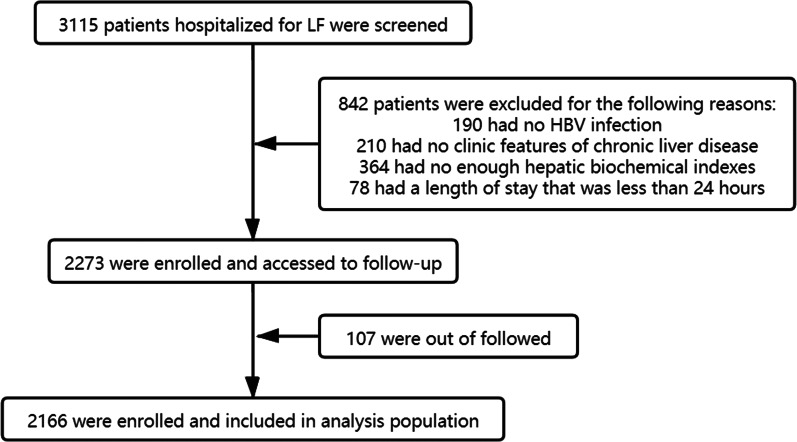
Flow diagram of the study subjects. A total of 3115 (from January 2010 to March 2018) liver failure patients were screened. Finally, 2166 patients qualified were follow-up for 90 days.

The flow chart for selecting patients with HBV-ACLF is shown in Figure 1.

### Identifying comorbidities and complications

Comorbidities included hepatic comorbidities (alcoholic liver disease, NAFLD, chronic hepatitis C virus, acute hepatitis E virus, autoimmune hepatitis (AIH), primary biliary cirrhosis (PBC), primary sclerosis cholangitis (PSC), Wilson's disease and cholelithiasis with surgery) and non-hepatic comorbidities (type 2 diabetes mellitus (T2DM), hyperthyroidism, CKD). Complications included spontaneous bacterial peritonitis, pneumonia and intestinal fungal infections, HE, gastrointestinal bleeding and HRS.

### Clinical data and information collection

Standardised case report forms were completed by trained physicians in all cases. The endpoint of our study was 90-day transplant-free mortality. The objective of our study was to find independent risk factors for survival in HBV-ACLF.

The study protocol was approved by the Clinical Trials Ethics Committee of the Third Affiliated Hospital of Sun Yat-sen University, and the requirement of written informed consent was waived. The study strictly adhered to the ethical guidelines of the Declaration of Helsinki and was registered with ClinicalTrials.gov (NCT03992898).

### Statistical analysis

All data were analysed using SPSS version 25.0. Data for clinical and biochemical characteristics were expressed as frequencies and percentages for categorical variables and mean ± standard deviation and medians (interquartile range) for continuous variables. The prevalence of comorbidities and complications (% and 95% confidence interval (CI)) was described. A chi-square test was used to compare frequencies. Baseline variables considered clinically relevant, or that showed a univariate relationship with the outcome were entered into a multivariate Cox proportional hazards regression model. The variables were judged using the Kaplan–Meier method, correlation tests for the Schoenfeld residuals and survival time ranks and time-dependent covariate tests to check the proportional hazards assumption. *P* values <0.05 were considered statistically significant.

## Results

### Demographic and clinical characteristics of ACLF patients

In this study, a total of 2166 patients with HBV-ACLF were enrolled between 1 January 2010 and 1 March 2018 (Table 1). As shown in the table, 1914 (88.37%) were male, and 678 (31.30%) were HBeAg positive.

**Table 1.** Demographic and clinical characteristics of 2166 enrolled patients

Characteristic	Number/scores
Gender, *n* (%)	
Male, *n* (%)	1914 (88.37)
Female, *n* (%)	252 (11.63)
Age, years	45.0 (37.0–54.0)
18–39, *n* (%)	718 (33.15)
40–59, *n* (%)	1113 (51.38)
⩾60, *n* (%)	335 (15.47)
WBC (/nl)	7.43 (5.68–9.84)
HGB (g/l)	121.00 (105.00–135.00)
PLT (/nl)	112.00 (76.00–152.00)
ALT (U/l)	324.00 (103.00–849.25)
ALB (g/l)	32.40 (29.50–38.00)
TBil (μmol/l)	371.90 (274.73–591.61)
Sodium (mmol/l)	137.00 (134.00–139.00)
Cr (μmol/l)	71.00 (61.00–85.00)
PT (s)	25.90 (22.10–31.90)
INR	2.36 (1.93–3.06)
HBeAg (+), *n* (%)	678 (31.30)
HBV-DNA loads (log IU/ml)	5.17 (3.59–6.71)
AFP (ng/ml)	50.30 (15.55–147.20)
Basics of liver disease, *n* (%)	
Chronic hepatitis B	858 (39.61)
Cirrhosis	1308 (60.39)
Liver cancer, *n* (%)	70 (3.23)
Child-Pugh score	11 (10.0–12.0)
A, *n* (%)	0 (0)
B, *n* (%)	393 (18.14)
C, *n* (%)	1773 (81.86)
MELD score	25.61 (22.68–29.81)
Comorbidity, *n* (%)	682 (31.49)
Complications at admission, *n* (%)	1300 (60.02)
Complications during hospitalisation, *n* (%)	1701 (78.53)
NAs therapy before the onset, *n* (%)	340 (15.70)
Received anti-HBV therapy, *n* (%)	1984 (91.60)
Liver transplantation	129 (5.96)
Mortality	1026 (47.37)

WBC, white blood count; HGB, haemoglobin; PLT, platelet; ALT, alanine transaminase; ALB, albumin; TBil, total bilirubin; Cr, creatine; PT, prothrombin time; INR, international normalised ratio; AFP, *α*-fetoprotein; MELD score, model for end-stage liver disease.

Data were expressed as median (interquartile range, IQR), or *n* (%). *P* values were calculated by Mann–Whitney *U* test, *χ*^2^ test, as appropriate.

Of these patients, 1308 (60.39%) were diagnosed with cirrhosis. Six hundred and eighty-two (31.49%) patients had one or more comorbidities. Thirteen hundred (60.02%) patients had at least one complication at the time of admission. During hospitalisation, the incidence of complications increased to 1701 (78.53%).

Three hundred and forty (15.70%) patients started nucleotide analogues (NAs) for more than 6 months prior to the onset of liver failure and experienced discontinuation or resistance to NAs. Antiviral drugs included lamivudine, adefovir dipivoxil, telbivudine, entecavir, tenofovir or a combination were used for at least 6 months. One thousand nine hundred and eighty-four (91.60%) patients received anti-HBV therapy after admission to the hospital. The 90-day survival rate for these patients who received NAs was 51.71%, compared with 30.77% for those who refused NAs.

One-hundred and twenty-nine (5.96%) patients needed and received liver transplantation. The 90-day transplant-free mortality rate in our study group was 47.37%.

### Prevalence of comorbidities

**Fig. 2.**
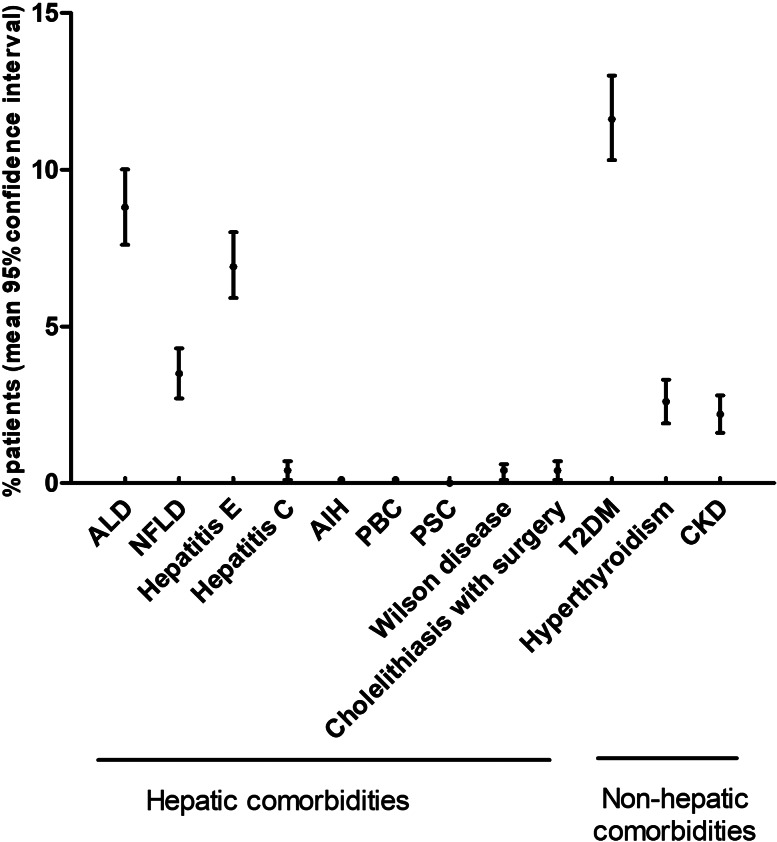
Prevalence of evaluated comorbidities in patients. ALD, alcoholic liver disease; NFLD, nonalcoholic fatty liver disease; AIH, autoimmune hepatitis; PBC, primary biliary cirrhosis; PSC, primary sclerosis cholangitis; T2DM, type 2 diabetes mellitus; CKD, chronic kidney disease.

The prevalence of comorbidities is depicted in Figure 2. We analysed the comorbidities of the patients and found that T2DM was the most common comorbidity (11.6%, 95% CI 10.3–13), followed by alcoholic liver disease (8.8%, 95% CI 7.6–10). Some other diseases such as AIH, PBC, PSC and Wilson's disease were rare in patients with HBV-ACLF, with a prevalence of <1% (Supplementary Table S1).

### The incidence of complications

**Fig. 3.**
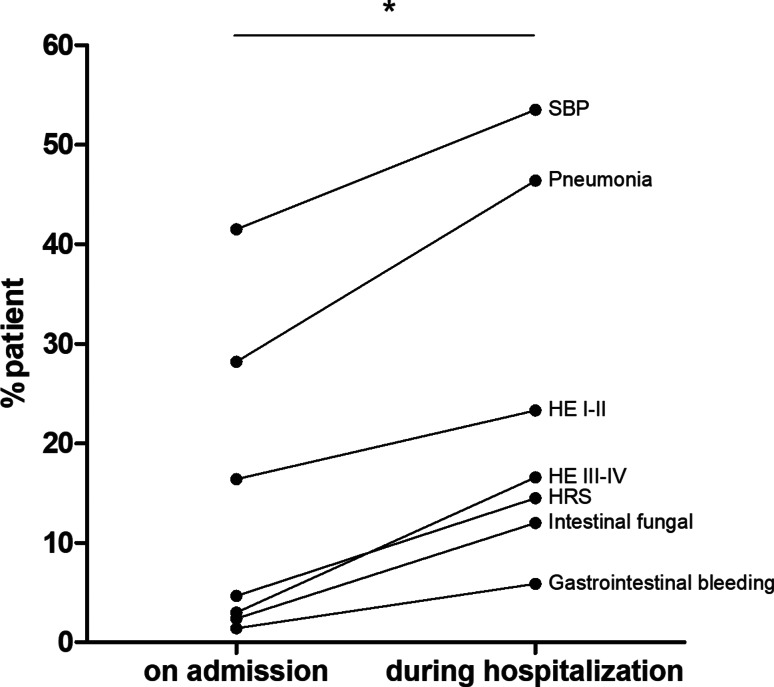
Incidence of evaluated complications in patients. SBP, spontaneous bacterial peritonitis, HE, hepatic encephalopathy, HRS, hepatorenal syndrome. *P < 0.001.

The incidence of complications is shown in Figure 3. We analysed reported complications such as infections (spontaneous bacterial peritonitis, pneumonia and intestinal fungal infections), gastrointestinal bleeding, HE and HRS on admission and during hospitalisation. On admission, the most common complication was infection. Spontaneous bacterial peritonitis topped the list (41.5%, 95% CI 39.4–43.5), followed by pneumonia (28.2%, 95% CI 26.3–30.1). Gastrointestinal bleeding was the least reported complication (1.4%, 95% CI 0.9–1.9). Most reported HE was found to be mild to moderate (grade I or II HE) (16.4%, 95% CI 14.9–18.0). The incidence of complications increased significantly (*P* < 0.001) during hospitalisation (Supplementary Table S2).

### Risk factors for the mortality of patients with HBV-ACLF

Cox regression analyses were performed to examine whether comorbidities and complications were associated with mortality in patients with HBV-ACLF. A series of proportional hazards regression analyses are summarised in Table 2 and Supplementary Table S3. After adjustment, the multivariable analysis revealed that age, white cell counts, HBV-DNA loads, cirrhosis, liver cancer, Child-Pugh grade, MELD scores and discontinuation of NAs were risk factors for increased 90-day mortality, while alpha-fetoprotein (AFP) was associated with a reduced mortality rate. T2DM was associated with increased mortality risk (unadjusted hazard ratio (HR) 1.512, 95% CI 1.273–1.796, adjusted HR 1.266, 95% CI 1.051–1.524) and hyperthyroidism (adjusted HR 1.893, 95% CI 1.312–2.732). Regarding complications, patients with gastrointestinal bleeding on admission (unadjusted HR 2.560, 95% CI 1.692–3.872, adjusted HR 1.809, 95% CI 1.173–2.791), HE on admission (unadjusted HR 3.055, 95% CI 2.673–3.492, adjusted HR 2.151, 95% CI 1.849–2.501), HE during hospitalisation (unadjusted HR 5.089, 95% CI 4.465–5.801, adjusted HR 3.197, 95% CI 2.761–3.703) and HRS during hospitalisation (unadjusted HR 3.406, 95% CI 2.961–3.918, adjusted HR 1.324, 95% CI 1.121–1.564) were found to have increased risk for the 90-day mortality due to ACLF.

**Table 2.** Multivariable analysis of risk factors for 90-day mortality

	Multivariate HR (95% CI)	*P*
Gender (male)	1.056 (0.859–1.298)	0.606
Age	1.028 (1.022–1.033)	0.000
WBC	1.033 (1.018–1.048)	0.000
HGB	1.003 (0.999–1.006)	0.142
PLT	0.999 (0.998–1.000)	0.120
ALT	1.000 (1.000–1.000)	0.422
ALB	0.998 (0.984–1.014)	0.804
Sodium	0.993 (0.984–1.001)	0.097
HBeAg (+)	0.884 (0.757–1.033)	0.120
HBV-DNA loads	1.120 (1.080–1.161)	0.000
AFP	0.999 (0.999–1.000)	0.004
Cirrhosis	1.310 (1.108–1.549)	0.002
Liver cancer	2.096 (1.565–2.808)	0.000
Child-Pugh grade (C)	1.460 (1.168–1.826)	0.000
MELD score	1.103 (1.093–1.114)	0.000
Discontinuation to NAs	1.359 (1.148–1.609)	0.000
Hyperthyroidism	1.893 (1.312–2.732)	0.001
T2DM	1.266 (1.051–1.524)	0.013
CKD	0.941 (0.656–1.350)	0.742
Alcoholic liver disease	0.942 (0.753–1.178)	0.600
Hepatitis E virus	0.819 (0.629–1.067)	0.139
NAFLD	1.156 (0.795–1.681)	0.446
Complication at admission		
SBP	1.098 (0.959–1.258)	0.177
Pneumonia	1.108 (0.957–1.283)	0.169
Intestinal fungal infection	0.808 (0.543–1.203)	0.293
Gastrointestinal bleeding	1.809 (1.173–2.791)	0.007
HE	2.151 (1.849–2.501)	0.000
I–II	1.985 (1.694–2.326)	0.000
III–IV	4.333 (3.104–6.049)	0.000
HRS	0.911 (0.695–1.193)	0.499
Complication during hospitalisation		
SBP	1.088 (0.941–1.257)	0.256
Pneumonia	1.007 (0.875–1.158)	0.926
Intestinal fungal infection	0.866 (0.713–1.053)	0.149
Gastrointestinal bleeding	1.207 (0.960–1.517)	0.107
HE	3.197 (2.761–3.703)	0.000
I–II	2.410 (2.046–2.840)	0.000
III–IV	5.530 (4.634–6.600)	0.000
HRS	1.324 (1.121–1.564)	0.001

WBC, white blood count; HGB, haemoglobin; PLT, platelet; ALT, alanine transaminase; ALB, albumin; TBil, total bilirubin; Cr, creatine; PT, prothrombin time; INR, international normalised ratio; AFP, *α*-fetoprotein; MELD score, model for end-stage liver disease; NAs, nucleotide analogues; SBP, spontaneous bacterial peritonitis, HE, hepatic encephalopathy, HRS, hepatorenal syndrome, HR, hazard ratio; CI, confidence interval.

Data were expressed as median (IQR), or *n* (%). *P* values were calculated by Mann–Whitney *U* test, *χ*^2^ test, as appropriate.

## Discussion

In this study, we investigated the clinical outcomes of Chinese patients with HBV-ACLF in a retrospective observational study. We initially explored whether various comorbidities and complications affect 90-day transplant-free survival. Our study showed that comorbidities and complications were common in these patients. Various complications frequently occurred during hospitalisation. T2DM and hyperthyroidism were risk factors for 90-day mortality, together with gastrointestinal bleeding and HE on admission and HE and HRS during hospitalisation.

In clinical practice, patients who have been diagnosed with ACLF often have many comorbidities such as T2DM, other hepatitis virus infections (e.g. hepatitis C and E) and alcoholic liver disease. In our study cohort, 31.49% of patients had one or more comorbidities. Berzigotti *et al*. found that the presence of comorbidities such as obesity, sarcopaenia and other metabolic risk factors such as diabetes mellitus, hypertension and dyslipidaemia impacted the prognosis of patients with cirrhosis [14]. Similarly, Duseja *et al*. found significantly higher MELD scores and increased mortality in overweight or obese patients [15]. A study by Mahmud *et al*. showed that the prevalence of diabetes mellitus, CKD and hepatocellular carcinoma in patients with ACLF of different aetiologies were 43%, 2.4% and 10.8%, respectively, while in our cohort, the prevalence was 11.49%, 2.22% and 3.23% [16]. These studies suggest that patients with HBV-ACLF commonly have a variety of comorbidities. These comorbidities may interact to influence the progression and prognosis of liver diseases.

Poorly controlled diabetes mellitus in people with liver disease can impact their recovery. Many studies have shown that effective control of hyperglycaemia can reduce complications and mortality in patients with diabetes complicated by chronic liver disease [17–19]. T2DM was the most common risk factor for mortality in our study. Its HR was 1.266 (95% CI 1.051–1.524). Hepatic insulin clearance is reduced in patients with liver failure, leading to an increase in glycosylation end products, hypoxia and hypoxia-induced factors, that reduce liver blood supply and inhibit liver regeneration [20], thus affecting the prognosis of patients.

Patients diagnosed with hyperthyroidism often have abnormal liver function tests that can lead to liver failure [21–23]. In our study, only 2.59% of the patients had hyperthyroidism. However, hyperthyroidism was a risk factor for mortality, with an adjusted HR of 1.893 (95% CI 1.312–2.732). It seems that due to the increased metabolic rate, the patient's oxygen consumption increased, which led to a relative hypoxia in the perivenous region, which in turn led to apoptosis and oxidative stress.

ACLF is a clinically recognised clinical syndrome characterised by severe liver injury and extrahepatic organ failure associated with short-term mortality. In the last few years, guideline reviews presented by the European Association for the Study of Liver (EASL) and the Asian Pacific Association for the Study of the Liver (APASL) have highlighted the importance of extrahepatic organ failure in the prevalence, characteristics, process and impact on the prognosis of ACLF [1, 24]. In our study cohort, we focused on some common complications in patients: abdominal, pulmonary and intestinal infections, gastrointestinal bleeding, HE and HRS. We noticed that 60.0% of the patients had at least one complication on admission, while 78.3% developed complications during their hospital stays. We also found that gastrointestinal bleeding and HE at admission, HE and HRS during hospitalisation were risk factors for 90-day mortality.

Due to the pathophysiological changes in ACLF, portal hypertension increases, contributing to the development of variceal bleeding. In our study, 1.39% of patients suffered gastrointestinal bleeding on admission, and 5.91% had gastrointestinal bleeding during hospitalisation. These data show a decrease in incidence compared to other studies, where the prevalence ranged from 15.6% to 31.2% [25–27]. The lower rates of variceal bleeding observed in our patients may be due to the lower prevalence of cirrhosis at the time of admission in our study population compared to previous studies. Patients with gastrointestinal bleeding on admission had increased mortality.

HE is associated with higher mortality, especially in patients with grades III and IV encephalopathy. The North American Consortium for the Study of End-Stage Liver Disease (NACSELD) showed that grades III and IV HE remains a significant determinant of 30-day mortality in patients hospitalised with cirrhosis, independent of other organ failures [10]. In the AARC data, HE was present in approximately 40% of patients with HBV-ACLF [28]. Several other studies have shown that the incidence of HE was 17.2–57.2% [10, 29]. In our study, 19.44% of patients had HE on admission, while 39.84% developed HE during hospitalisation. Among these hospitalised patients, 41.6% were diagnosed with HE III–IV. HE on admission and during hospitalisation was both risk factors for mortality.

HRS is a liver-specific functional renal failure associated with a high mortality rate. It is caused by a combination of marked activation of the sympathetic and neurohumoral systems and splanchnic vasodilatation, systemic hypotension and intense renal vasoconstriction [30]. Renal dysfunction has been reported in 22.8–34.0% of patients with ACLF [11, 29]. We found that only 4.71% of the patients had HRS on admission, while the incidence increased to 14.50% during hospitalisation. Furthermore, HRS during hospitalisation was a risk factor for 90-day mortality in ACLF.

Our study had several strengths. First, the sample size of this study was large, including patients between 2010 and 2018, which reflects the changes in HBV-ACLF treatment over the past decade. Second, we examined a range of patient-level variables that may affect mortality, including sociodemographic characteristics, laboratory indices, the severity of liver disease, comorbidities and complications. Third, we took comorbidities and complications together to evaluate the risk factors for mortality, which may better reflect the relationship between comorbidities, complications and mortality.

Our study also had several limitations. First, our patients were from a single-centre, and the genetic background and environmental variables (e.g. dietary habits) of the participants may not represent the entire population. Furthermore, this study was retrospective and observational, and some patients may not have had an oral glucose tolerance test to diagnose diabetes during hospitalisation, which may have led to a missed diagnosis of diabetes. In addition, we did not access the Hepatitis D virus test results and did not report alcohol consumption. As a result, this made it impossible to evaluate the impact of HDV on HBV-ACLF or potentially explain the high rates among males.

In conclusion, our results demonstrate that comorbidities and complications should be used to identify the prognosis of patients with HBV-ACLF who are at high risk of short-term mortality. Comorbidity assessment may accurately complement objective measures of liver disease severity and improve the care of those patients at high risk of death due to HBV-ACLF. It may also allow for a comprehensive health status assessment and better risk stratification of patients with HBV-ACLF. Our study provides the necessary data, including comorbidities and complications associated with mortality, to be considered candidate variables for future prognostic models.

## Data Availability

The data analysed during the current study are available from the corresponding author on request.

## Supplementary material

For supplementary material accompanying this paper visit http://doi.org/10.1017/S0950268822001169. click here to view supplementary material

## References

[1] Sarin SK (2019) Acute-on-chronic liver failure: consensus recommendations of the Asian Pacific association for the study of the liver (APASL): an update. Hepatology International 13, 353–390. 31172417 10.1007/s12072-019-09946-3 PMC6728300

[2] Garg H (2012) Clinical profile and predictors of mortality in patients of acute-on-chronic liver failure. Digestive and Liver Disease 44, 166–171. 21978580 10.1016/j.dld.2011.08.029

[3] Liu XY, Hu JH and Wang HF (2009) Analysis of prognostic factors for patients with acute-on-chronic liver failure. Chinese Journal of Hepatology 17, 607–610. 19719920

[4] Liu J (2019) Countdown to 2030: eliminating hepatitis B disease, China. Bulletin of the World Health Organization 97, 230–238. 30992636 10.2471/BLT.18.219469 PMC6453311

[5] Si J (2018) Chronic hepatitis B virus infection and risk of chronic kidney disease: a population-based prospective cohort study of 0.5 million Chinese adults. BMC Medicine 16, 93. 29909773 10.1186/s12916-018-1084-9 PMC6004660

[6] Du Y (2019) Association between hepatitis B virus infection and chronic kidney disease: a cross-sectional study from 3 million population aged 20 to 49 years in rural China. Medicine (Baltimore) 98, e14262. 30702585 10.1097/MD.0000000000014262 PMC6380805

[7] Pang Y (2018) Diabetes, plasma glucose, and incidence of fatty liver, cirrhosis, and liver cancer: a prospective study of 0.5 million people. Hepatology (Baltimore, MD) 68, 1308–1318. 10.1002/hep.30083 PMC6220764 29734463

[8] Joo EJ (2017) Hepatitis B virus infection and decreased risk of nonalcoholic fatty liver disease: a cohort study. Hepatology (Baltimore, MD) 65, 828–835. 10.1002/hep.28917 28035771

[9] Wong VW (2012) Hepatitis B virus infection and fatty liver in the general population. Journal of Hepatology 56, 533–540. 22027575 10.1016/j.jhep.2011.09.013

[10] Bajaj JS (2017) Hepatic encephalopathy is associated with mortality in patients with cirrhosis independent of other extrahepatic organ failures. Clinical Gastroenterology and Hepatology 15, 565–574.e4. 27720916 10.1016/j.cgh.2016.09.157

[11] Zang H (2016) Incidence, risk factors and outcomes of acute kidney injury (AKI) in patients with acute-on-chronic liver failure (ACLF) of underlying cirrhosis. Hepatology International 10, 807–818. 27485174 10.1007/s12072-016-9756-z

[12] Jepsen P (2015) Diabetes as a risk factor for hepatic encephalopathy in cirrhosis patients. Journal of Hepatology 63, 1133–1138. 26206073 10.1016/j.jhep.2015.07.007

[13] Liver Failure and Artificial Liver Group Chinese Society of Infectious Diseases, Chinese Medical Association (2018) Guideline for diagnosis and treatment of liver failure. Journal of Clinical Hepatology 11, 401–410.

[14] Berzigotti A (2011) Obesity is an independent risk factor for clinical decompensation in patients with cirrhosis. Hepatology 54, 555–561. 21567436 10.1002/hep.24418 PMC3144991

[15] Duseja A (2021) Impact of metabolic risk factors on the severity and outcome of patients with alcohol-associated acute-on-chronic liver failure. Liver International 41, 150–157. 32970356 10.1111/liv.14671

[16] Mahmud N (2019) Incidence and mortality of acute-on-chronic liver failure using two definitions in patients with compensated cirrhosis. Hepatology 69, 2150–2163. 30615211 10.1002/hep.30494 PMC6461492

[17] Porepa L (2010) Newly diagnosed diabetes mellitus as a risk factor for serious liver disease. Canadian Medical Association Journal 182, E526–E531. 20566726 10.1503/cmaj.092144 PMC2917963

[18] El-Serag HB and Everhart JE (2002) Diabetes increases the risk of acute hepatic failure. Gastroenterology 122, 1822–1828. 12055590 10.1053/gast.2002.33650

[19] Bjorkstrom K (2019) Risk factors for severe liver disease in patients with type 2 diabetes. Clinical Gastroenterology & Hepatology 17, 2769–2775.e4. 31009793 10.1016/j.cgh.2019.04.038

[20] Leclercq IA (2007) Insulin resistance in hepatocytes and sinusoidal liver cells: mechanisms and consequences. Journal of Hepatology 47, 142–156. 17512085 10.1016/j.jhep.2007.04.002

[21] Sousa Dominguez A (2015) Severe acute liver failure and thyrotoxicosis: an unusual association. Revista Espanola de Enfermedades Digestivas 107, 572–576. 10.17235/reed.2015.3607/2014 26176693

[22] Soleimanpour SA (2015) Fulminant liver failure associated with delayed identification of thyroid storm due to heterophile antibodies. Clinical Diabetes & Endocrinology 1, 1–5. 10.1186/s40842-015-0012-6 PMC4610393 26491542

[23] Hasosah M (2017) Neonatal hyperthyroidism with fulminant liver failure: a case report. Journal of Clinical and Diagnostic Research 11, SD01–SSD2. 10.7860/JCDR/2017/21503.9641 PMC5449869 28571223

[24] European Association for the Study of the Liver. (2017) EASL clinical practical guidelines on the management of acute (fulminant) liver failure. Journal of Hepatology 66, 1047–1081. 28417882 10.1016/j.jhep.2016.12.003

[25] Mucke MM (2018) Bacterial infection-triggered acute-on-chronic liver failure is associated with increased mortality. Liver International 38, 645–653. 28853199 10.1111/liv.13568

[26] Cao Z (2019) The impact of HBV flare on the outcome of HBV-related decompensated cirrhosis patients with bacterial infection. Liver International 39, 1943–1953. 31206235 10.1111/liv.14176

[27] Zhao H (2019) Upper gastrointestinal hemorrhage in acute-on-chronic liver failure: prevalence, characteristics, and impact on prognosis. Expert Review of Gastroenterology and Hepatology 13, 263–269. 30791764 10.1080/17474124.2019.1567329

[28] Choudhury A (2017) Liver failure determines the outcome in patients of acute-on-chronic liver failure (ACLF): comparison of APASL ACLF research consortium (AARC) and CLIF-SOFA models. Hepatology International 11, 461–471. 28856540 10.1007/s12072-017-9816-z

[29] Tong JJ (2019) Predictive value of the Chinese group on the study of severe hepatitis B-acute-on-chronic liver failure score in the short-term prognosis of patients with hepatitis B virus-related acute-on-chronic liver failure. Chinese Medical Journal 132, 1541–1549. 31188162 10.1097/CM9.0000000000000298 PMC6616238

[30] Bernal W (2015) Acute-on-chronic liver failure. The Lancet (London, England) 386, 1576–1587. 10.1016/S0140-6736(15)00309-8 26423181

