# Supplementary material for: Risk factors for underlying comorbidities and complications in patients with hepatitis B virus-related acute-on-chronic liver failure
Source: Epidemiol Infect. 2022 Jul 5;150:e147. doi: 10.1017/S0950268822001169 (PMC9354478; doi:10.1017/S0950268822001169)
Supplement: Supplementary file 1 [file S0950268822001169sup001.docx]

Supplementary Table 1

Supplementary Table 1: Underlying comorbidities of all patients

| Comorbidity | ICD-10 code | Prevalence, n (%) |
| --- | --- | --- |
| Hyperthyroidism | E05.803 | 56 (2.59) |
| Type 2 Diabetes Mellitus | E11.900 | 252 (11.49) |
| Chronic kidney disease | N18.x | 48 (2.22) |
| Alcoholic liver disease | K70.x | 191 (8.82) |
| Nonalcoholic fatty liver disease | K76.001 | 76 (3.51) |
| Hepatitis C virus | B18.251 | 9 (0.42) |
| Hepatitis E virus | B17.x | 150 (6.93) |
| Autoimmune hepatitis | K75.4 | 2 (0.09) |
| Primary biliary cirrhosis | K74.3 | 2 (0.09) |
| Primary  sclerosis cholangitis | K83.01 | 0 (0.00) |
| Wilson disease | E83.01 | 8 (0.37) |
| Cholelithiasis  with surgery | K80.x | 9 (0.42) |

All results were expressed as a percentage.

Supplementary Table 2

Supplementary Table 2: The incidence of complications of patients on admission and during hospitalization

| Complication | On admission, n (%) | During hospitalization, n (%) | P |
| --- | --- | --- | --- |
| SBP | 898 (41.46) | 1159 (53.51) | 0.000 |
| Pneumonia | 611 (28.21) | 1004 (46.35) | 0.000 |
| Intestinal fungal infection | 51 (2.35) | 260 (12.00) | 0.000 |
| Gastrointestinal bleeding | 30 (1.39) | 128 (5.91) | 0.000 |
| Hepatic encephalopathy | 421 (19.44) | 863 (39.84) | 0.000 |
| I-II | 356 (16.44) | 504 (23.27) | 0.000 |
| III-IV | 65 (3.00) | 359 (16.57) | 0.000 |
| Hepatorenal syndrome | 102 (4.71) | 314 (14.50) | 0.000 |

All results were expressed as a percentage.

SBP: Spontaneous bacterial peritonitis

Supplementary Table 3

Supplementary Table 3: Univariable analysis of risk factors for 90-day mortality

|  | Univariate  HR (95% CI) | P |
| --- | --- | --- |
| Gender (male) | 0.881 (0.731-1.062) | 0.185 |
| Age | 1.038 (1.033-1.043) | 0.000 |
| WBC | 1.058 (1.045-1.073) | 0.000 |
| HGB | 0.992 (0.990-0.995) | 0.000 |
| PLT  ALT | 0.997 (0.996-0.998)  1.000 (1.000-1.000) | 0.000  0.910 |
| ALB | 0.958 (0.944-0.971) | 0.000 |
| Sodium | 0.985 (0.980-0.989) | 0.000 |
| HBeAg (+) | 0.766 (0.667-0.879) | 0.000 |
| HBV-DNA loads | 1.04 (1.009-1.073) | 0.013 |
| AFP | 0.999 (0.998-0.999) | 0.000 |
| Cirrhosois | 1.978 (1.725-2.268) | 0.000 |
| liver cancer | 2.404 (1.843-3.136) | 0.000 |
| Child-Pugh grade (C) | 2.396 (1.959-2.930) | 0.000 |
| MELD score | 1.115 (1.107-1.124) | 0.000 |
| Previous antiviral therapy | 1.472 (1.257-1.724) | 0.000 |
| Hyperthyroidism | 1.371 (0.964-1.949) | 0.079 |
| Type 2 Diabetes Mellitus | 1.512 (1.273-1.796) | 0.000 |
| Chronic kidney disease | 1.937 (1.382-2.714) | 0.000 |
| Alcoholic liver disease | 0.958 (0.772-1.188) | 0.695 |
| Hepatitis E virus | 0.892 (0.695-1.144) | 0.368 |
| Nonalcoholic fatty liver disease | 9.832 (0.582-1.189) | 0.312 |
| Complication at admission |  |  |
| SBP | 1.664 (1.472-1.881) | 0.000 |
| Pneumonia | 1.789 (1.575-2.032) | 0.000 |
| Intestinal fungal infection | 1.171 (0.804-1.705) | 0.410 |
| Gastrointestinal bleeding | 2.560 (1.692-3.872) | 0.000 |
| HE | 3.055 (2.673-3.492) | 0.000 |
| I-II | 2.857 (2.478-3.295) | 0.000 |
| III-IV | 4.743 (3.568-6.306) | 0.000 |
| HRS | 3.897 (3.137-4.842) | 0.000 |
| Complication during hospitalization |  |  |
| SBP | 1.965 (1.727-2.237) | 0.000 |
| Pneumonia | 1.921 (1.696-2.176) | 0.000 |
| Intestinal fungal infection | 0.941 (0.781-1.134) | 0.521 |
| Gastrointestinal bleeding | 1.954 (1.580-2.417) | 0.000 |
| HE | 5.089 (4.465-5.801) | 0.000 |
| I-II | 3.631 (3.126-4.219) | 0.000 |
| III-IV | 9.161 (7.850-10.691) | 0.000 |
| HRS | 3.406 (2.961-3.918) | 0.000 |

Data were expressed as median (Interquartile Range, IQR), or n (%). p values were calculated by Mann–Whitney U test, χ2 test, as appropriate.

WBC: white blood count; HGB: hemoglobin; PLT: platelet; ALT: alanine transaminase; ALB: albumin; TBil; total bilirubin; Cr: creatine; PT: prothrombin time; INR: international normalized ratio; AFP: α-fetoprotein; MELD score: Model for end-stage liver disease; NAs: nucleotide analogs; SBP: Spontaneous bacterial peritonitis, HE: Hepatic encephalopathy, HRS: Hepatorenal syndrome, HR: Hazard ratio; CI: confidence interval
